# Asymmetrical Polyhedral Configuration of Giant Vesicles Induced by Orderly Array of Encapsulated Colloidal Particles

**DOI:** 10.1371/journal.pone.0146683

**Published:** 2016-01-11

**Authors:** Yuno Natsume, Taro Toyota

**Affiliations:** 1 Department of Mathematical and Physical Sciences, Faculty of Science, Japan Women's University, Tokyo, Japan; 2 Research Center for Complex Systems Biology, The University of Tokyo, Tokyo, Japan; 3 Department of Basic Science, Graduate School of Arts and Sciences, The University of Tokyo, Tokyo, Japan; University of Helsinki, FINLAND

## Abstract

Giant vesicles (GVs) encapsulating colloidal particles by a specific volume fraction show a characteristic configuration under a hypertonic condition. Several flat faces were formed in GV membrane with orderly array of inner particles. GV shape changed from the spherical to the asymmetrical polyhedral configuration. This shape deformation was derived by entropic interaction between inner particles and GV membrane. Because a part of inner particles became to form an ordered phase in the region neighboring the GV membrane, free volume for the other part of particles increased. Giant vesicles encapsulating colloidal particles were useful for the model of “crowding effect” which is the entropic interaction in the cell.

## Introduction

Living cells perform a variety of essential functions, (e.g., signal transduction, component configuration, efficient transport, and energy conversion) with far less energy than that required by man-made machines or semiconductors of the comparable size. Being one of the mechanisms realizing such functions in living cells, the “crowding effect” has garnered much attention. This effect is induced by high concentrations of macromolecules inside the cells [1, 2]. It has the following two effects; reduces the diffusion rate, and increases the free volume for macromolecules [1]. The free volume for macromolecule is defined as the volume not occupied by the macromolecules and available for their movement as possible as their centers of mass can approach [2]. The free volume is thus restricted by both crowded medium and boundary. Previous studies found that the folding of proteins or the formation of macromolecules and their complexes are induced by the effect of increasing free volume [3–5]. Furthermore, it was proposed that hypertonic condition of inner molecules was involved in shape deformation of liposomes experimentally [6]. Since the effect of increasing free volume is based on entropy change, this effect is independent of the composition of a substance. Therefore, bottom-up approaches of modeling are highly applicable to investigation of cell functions in terms of the crowding effect. For example, when DNA and alginate were confined inside water-in-oil emulsions under highly condensed conditions, DNA was localized on the surface of the emulsion droplets [7]. Spherical giant vesicles (GVs) containing polystyrene beads or polyethylene glycols at high densities (concentrations) tend to divide into a couple of small GVs [8, 9]. This division is plausibly induced by maximization of the free volume for such intentional particles and molecules.

We previously established a method for preparing GVs involving hard spherical particles of micrometer size with various volume fractions [10]. This experimental model has two advantages. One is direct evaluation of the volume fraction of each GV by counting fluorescent particles under a fluorescence microscope. The other is visualization of the distribution of particles and the state of arrangement inside the GVs. Here, we prepared GVs confining fluorescent polystyrene beads of 1 μm in diameter for visualizing by fluorescent microscopy. Next, we added a hypertonic solution to the GVs and found that part of the internal particles exhibited an ordered array near the membrane. Moreover, in association with this characteristic arrangement of the internal particles, a part of the GV membrane turned angular in shape, and the GV exhibited shape deformation to asymmetrical polyhedral configuration. Consequently, we proposed a model based on the equivalence of osmotic pressures of internal particles in both ordered and disordered phases, considering the free volume for the particles inside the GV under such geometrical conditions. This model led us to the convincing result of the experimental findings on the volume fraction of internal particles and the GV size. Namely, it was successfully indicated that the asymmetrical polyhedral configuration of the GV is derived from the entropic effect of the internal particles which, in the disordered phase, increase their free volume and make array in the vicinity of GV membrane in the ordered phase.

## Results

**Fig 1:**
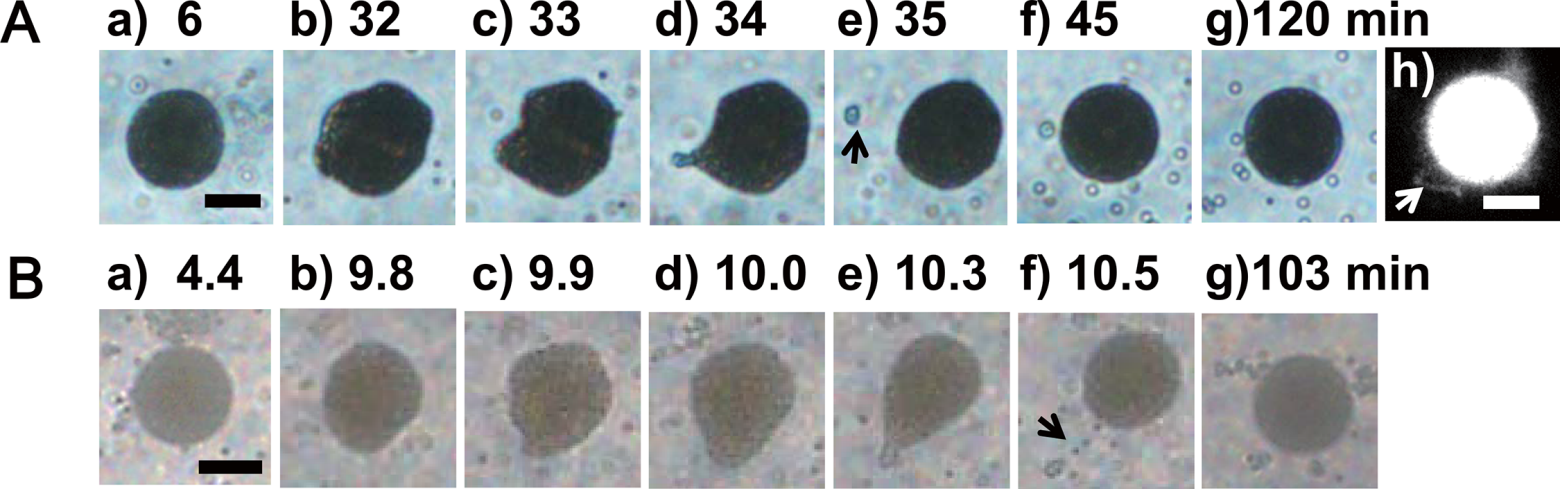
Time-dependence of shape deformation of GV encapsulating charged polystyrene beads at specific volume fraction. Phase contrast microscopy images of the time-dependent changes after addition of a hypertonic _D_-glucose solution. A: GV with diameter of 19 μm encapsulating charged polystyrene beads at 13 vol%. B: GV with diameter of 17 μm encapsulating charged polystyrene beads at 14 vol%. Microscopy images of (a)-(c) show the formation of angular regions of GV membrane. In contrast, (d)-(g) show the formation of tubular structure and diminishment of the angular regions. The fluorescence microscopy image of the GV (in A) 120 mins after addition is shown in (h). The black arrows in A (e) and B (f) and the white arrow in A (h) indicates the tubular structure attached to the GV. Scale bar = 10 μm.

The GVs confining polystyrene beads were prepared by the water-in-oil emulsion transfer method using centrifugation [10–12]. The phospholipid comprising the GVs was 1,2-dioleoyl-*sn*-glycero-3-phosphocholine (DOPC), while TexasRed®-tagged 1,2-dihexadecanoyl-*sn*-glycero-3-phosphoethanolamine triethylammo-nium salt (TexasRed® DHPE) was used as the membrane fluorescent dye (see METHODS, Materials, Preparation of GVs). In the present system of a GV confining polystyrene beads, the beads had a surface zeta potential of – 62 mV. Because inner water phase of GV was buffer solution, electrostatic interaction of polystyrene beads and GV membrane could be ignored. We added hypertonic aqueous solution (35 μL) of 1.1 M _D_-glucose to 5 μL of the prepared GV dispersion, as a result, the concentration difference of sugar between inner and outer water phases of the GV became 0.09 M. Immediately after the hypertonic aqueous solution was added to a dispersion of the prepared GVs in a chamber, the GVs were observed under a phase contrast and fluorescence microscope. In this study, we discuss the transformed GVs, which were ~30% of the GVs selected randomly (N = 28/92) in a batch of the chamber. The shape deformation of the GVs depended on the volume fraction of inner particles by estimating volume fractions at each GVs (see METHODS, Estimation of volume fraction). As shown in S1A and S1B Fig, the GVs containing polystyrene beads in volume fractions less than 10 vol% underwent transformation from sphere to either prolate/oblate morphology or budding during the first 20–90 mins following addition of the hypertonic solution. In case of GVs with its diameter of ca. 10 μm and volume fraction of 23 vol%, fission of GVs was observed (see S1C Fig). Geometrical relationship and size ratio of the GVs formed through such fission were almost equal to those of GVs, confining colloidal particles with volume fraction of ca. 50 vol%, in the previous study [8],. As a particular instance of intermediate volume fraction the GVs with diameter of ca. 20 μm and volume fraction of ca. 13 vol%, flat surfaces and multiple angular regions began to form gradually on the spherical surface a few dozen minutes after addition of the hypertonic solution (Fig 1A and 1B; a–c). This was followed, a few minutes later, by a prominent protrusion of the membrane in one region (Fig 1A and 1B; d), and transformation of this protrusion to a tubular structure. This was in turn followed by diminishment and disappearance of the angular regions on the membrane surface (Fig 1A and 1B; e). At last, the GVs formed spheres with attached tubular structures (Fig 1A and 1B; f-g, and 1A; h). In the case of GVs with volume fractions less than 10 vol%, the prolate or oblate conformation remained stable till the end of the temporal observation of 120 mins following addition of the hypertonic solution.

**Fig 2:**
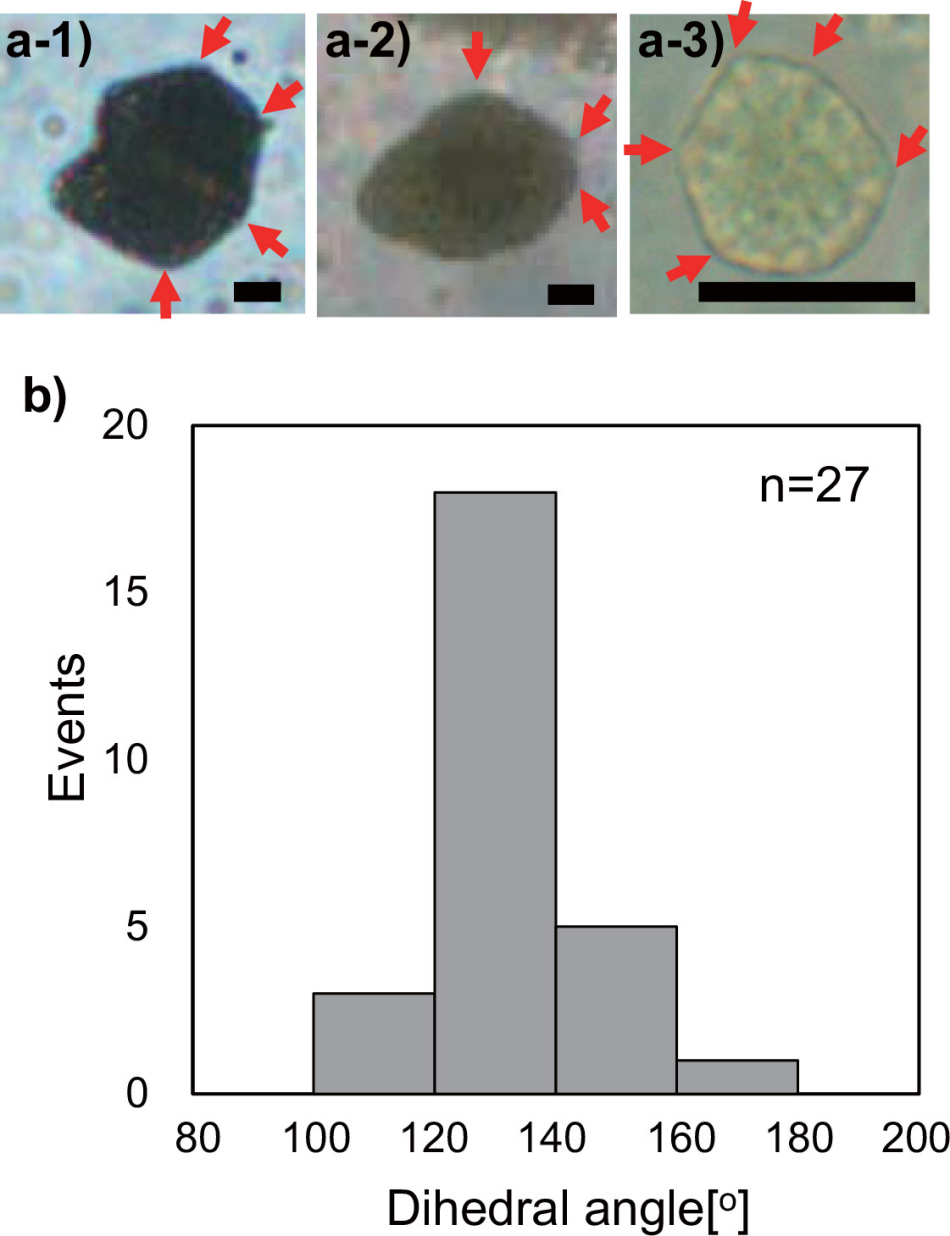
Dihedral angles of transformed GVs with angular surfaces. Phase contrast microscopy images of transformed GVs with angular surfaces (a), and histogram of dihedral angle occurrences (b). The points of dihedral angle measurement are indicated by red arrows in panel (a). Scale bar = 5 μm.

**Fig 3:**
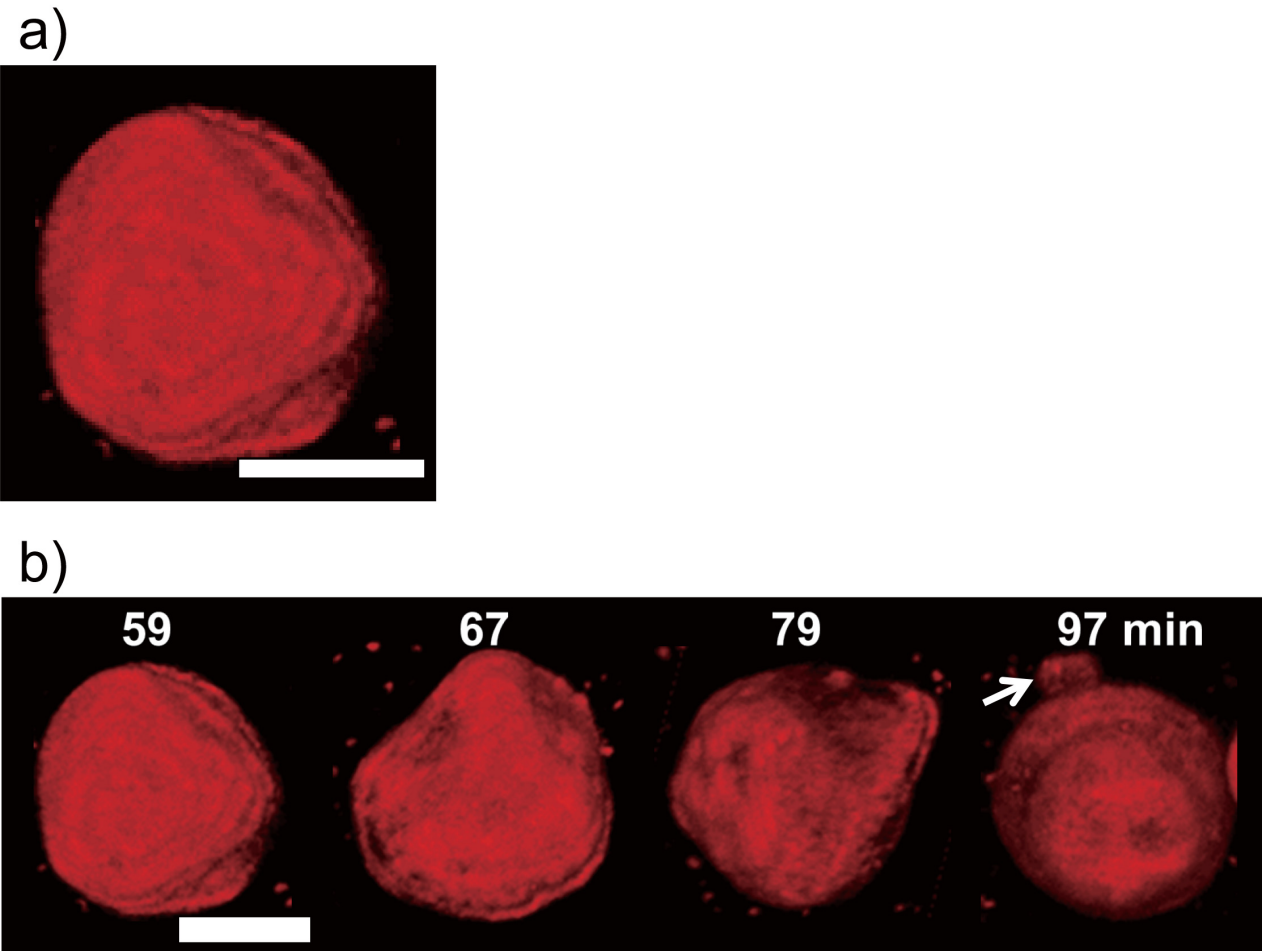
3-Dimensional images of the asymmetrical polyhedral GV constructed by the confocal laser scanning fluorescence microscopy images. Confocal laser scanning fluorescence microscopy images of asymmetrical polyhedral GV configuration and its temporal transition to a stable form. (a) Polyhedral GV image and schematic. (b) Temporal transition from asymmetrical polyhedral GV to stable form at 59, 67, 79, and 97 mins after addition of 1.1 M D-glucose solution. The white arrow indicates the tubular structure attached to the GV. Scale bar = 5 μm.

We observed such characteristic configuration with angular regions in GVs with the diameter of not only ca. 20 μm (Fig 2A-1,2) but also ca. 10 μm (Fig 2A-3). We measured the angles of the projection microscopy image of this GV configuration obtained under an epi-illuminance (indicated by the red arrows shown in Fig 2A) for estimation of dihedral angles of the membrane. In seven GVs whose diameters were 6.8–19 μm, the angular regions other than that of the protrusion part had obtuse angles in range of 100–180 degree. Following this, we constructed a histogram of the occurrence of various angles in the resulting polyhedral GVs (Fig 2B). The histogram shows the relatively narrow distribution with a single peak at ~ 130 degree.

In order to elucidate the precise GV configuration, we used a high spatial-resolution confocal laser scanning fluorescence microscope to obtain sequential fluorescent cross-sectional images of the fluorescent GV while scanning in the direction of the Z-axis. We also used software to derive the three-dimensional (3D) structure from these images (Fig 3). Fifty nine minutes after addition of the hypertonic solution, we observed flat triangular faces on several sides of the GV (Fig 3A). The 3D image constructed clearly indicated that the configuration of the GV is similar to part of a polyhedron which has triangular faces. Here, we named this characteristic GV as “asymmetrical polyhedral GV.” Fig 3B shows the constructed 3D images of the temporal transition in GV structure following its polyhedral formation 59 mins post addition of the hypertonic solution. At 79 mins, the images showed a protrusion developing from one region of the GV membrane (Fig 3B). At 97 mins, the images clearly showed its transformation into a spherical GV with an attached tube-like structure (Fig 3B).

**Fig 4:**
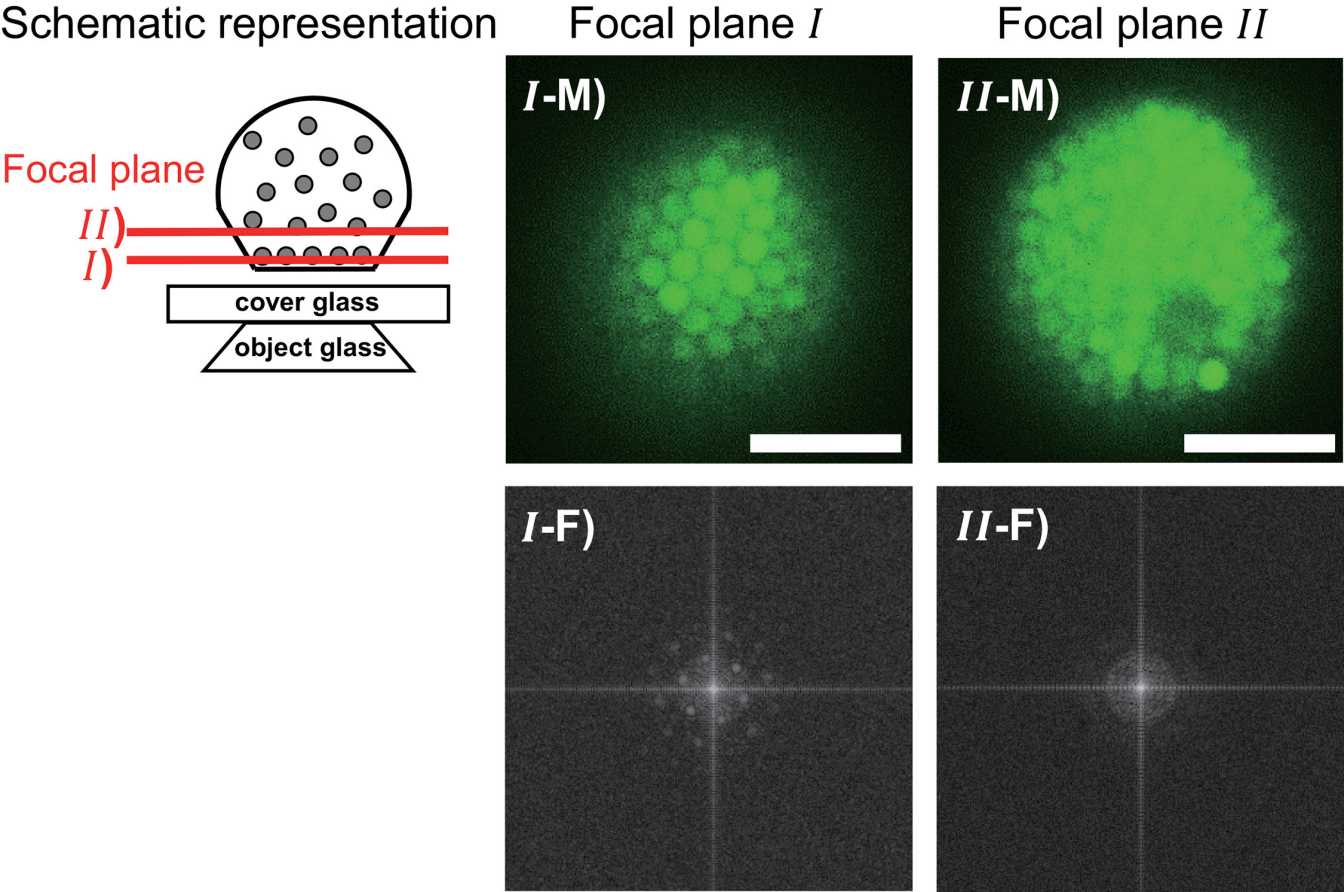
Ordered and disordered phases of particles confined in the asymmetrical polyhedral GV. Confocal laser scanning fluorescence microscopy images of asymmetrical polyhedral GV cross-sections at levels I and II, and their Fourier transformations. I-M, confocal microscopy image adjacent to GV base. I-F, Fourier transformation of that image. II-M, confocal microscopy image 2.2 μm from the GV base. II-F, Fourier transformation of that image. Scale bar = 5 μm.

Next, to investigate the particle distribution within the asymmetrical polyhedral GV, we used a high temporal resolution Nipkow-disk confocal laser scanning fluorescence microscope, and obtained multiple cross-sectional images of the internal fluorescent polystyrene beads in the same asymmetrical polyhedral GV. We found that the velocity of the internal particle in the asymmetrical polyhedral GV was smaller than those in non-deformed GV with low volume fraction, i.e. the movement of internal particles was restricted in the vicinity of the membrane of the asymmetrical polyhedral GV (S2 Fig). As shown in Fig 4, we further analyzed the fluorescence images of two cross sections (I and II) of the asymmetrical polyhedral GV, which, if spherical, would be 14 μm in diameter. Fig 4I-M and 4II-M are confocal images obtained during the initial microscope scans in the Z-axis direction, showing the particle groups adjacent to the GV base and 2.2 μm in the Z-axis direction from the GV base, respectively. From the microscopy images, we estimated that the interparticle distance between the polystyrene beads was 1.10 ± 0.04 μm in the region adjacent to the GV base. However, we observed interparticle gaps of 2.4 μm in the internal cross-section just 2.2 μm from the GV membrane. Fig 4I-F and 4II-F show the images obtained by two-dimensional Fourier transformation of the microscopy images using the ImageJ software [13]. The transformed image of the particles adjacent to the GV base membrane (Fig 4I-F) clearly shows points at a rotation of 60° from each particle position, thus indicating that the particles are organized in a regular hexagonal lattice array. In the cross-section at 2.2 μm from the GV base membrane, in contrast, the transformed image (Fig 4II-F) shows no points representing an orderly particle array, thus indicating that the particle positions are irregular. These results show that two particle distributions coexisted within the asymmetrical polyhedral GV; one with polystyrene beads in the region neighboring the GV membrane in an orderly array, and the second in other regions distributed irregularly.

## Discussion

Here we propose a model for the asymmetrical polyhedral GV based on the equivalence of osmotic pressures of internal particles in both ordered and disordered phases. Considering the free volume for the internal particles under such geometrical conditions, we calculate the volume fraction of internal particles and the GV size for the appearance of the configuration of GVs.

**Fig 5:**
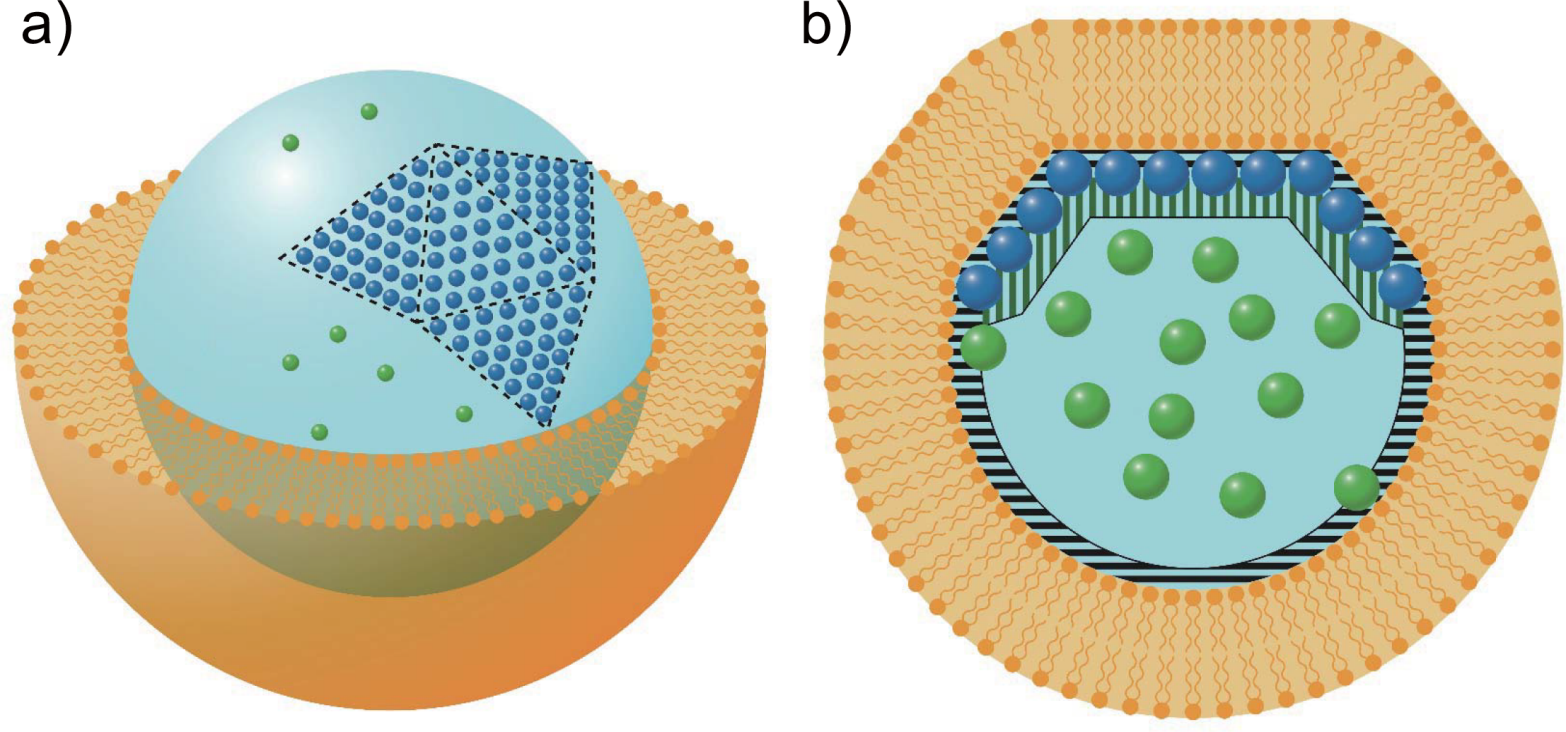
Schematic illustration of the asymmetrical polyhedral GV with depletion volume. (a) Schematic illustration of the asymmetrical polyhedral GV, which is constructed by four triangular surfaces of an icosahedron and a part of its circumscribing sphere. Blue and green balls show particles of ordered and disordered phases, respectively. (b) Schematic illustration of depletion volume of particles in the cross-section of (a). Black and horizontal and green vertical bars indicate the depletion volumes in the vicinity of membrane surface and around the surface of the particle group in the ordered phase, respectively.

At the beginning, the configuration was geometrically validated by the changes of volume and surface area of GV during the transformation (See METHODS, Modeling the shape of asymmetrical polyhedral GV). Fig 3A shows the presence of more than three flat triangular surfaces on the asymmetrical polyhedral GV. By using number *n* of triangular surfaces and the surface area change under shape deformation, we assumed that the asymmetrical polyhedral GV is constructed by four triangular surfaces of an icosahedron and a part of its circumscribing sphere as shown in Fig 5A.

We thus propose a model for the states in which both ordered and disordered phases appear inside the asymmetrical polyhedral GV configurations. The coexistence of the ordered and disordered phases occurs when both pressures and chemical potentials of the particles in both the phases become equal. As long as both phases are proximal to each other inside the GV, the chemical potentials are the same. Osmotic pressure *π*, which depends on the compressibility *ζ* of hard particles is important here. In fact, the free energy *F* of a colloidal particle system can be expressed as *F* = −*∫ πdV* using *π*. The relation between *π* and *ζ* is: $\pi =\frac{N}{V^{f}}k_{B}T\zeta$, where *N*, *V*^*f*^, *k*_B_, and *T* are the number of particles, the free volume for particles, a Boltzmann constant, and temperature, respectively (See METHODS, Osmotic balance in asymmetrical polyhedral GV). Therefore, for realizing coexistence of the two phases, *π*_*dis*_ = *π*_*ord*_, i.e., $\frac{V_{dis}^{f}}{V_{ord}^{f}}=\frac{N_{dis}}{N_{ord}}\times \frac{\zeta_{dis}}{\zeta_{ord}}$ is requisite. The subscripts “*dis*” and “*ord*” indicate the disordered and ordered phases, respectively. *ζ*_*dis*_ and *ζ*_*ord*_ are given by volume fractions *Φ*_*dis*_ and *Φ*_*ord*_ [14,15], respectively. In detail, $V_{dis}^{f}/V_{ord}^{f}\left(=\theta \right)$ is given by two parameters: radius, *R*_*s*_; and volume fraction, *Φ* in the initial spherical GV.

For geometrical consideration, *Rs* corresponds to the radius of the circumscribed sphere of the asymmetrical polyhedral GV (See METHODS, Modeling the shape of asymmetrical polyhedral GV). With regard to the other approach, $V_{dis}^{f}/V_{ord}^{f}$ was evaluated using geometric conditions based on the free volume for the encapsulated hard particles (See METHODS, Geometrical derivation on free volume for inner particles). Since the encapsulated particles were hard spheres, the center of mass cannot get close to GV membrane [8]. The depletion area in the vicinity of the GV membrane is depicted by black horizontal bars in Fig 5B. The free volume for the particles equals the GV volume subtracted from the volume of this depletion area (depletion volume). In this free volume, translation motion of particles has no restriction. In addition to this depletion area, the depletion area for particles in the disordered phase comes from the surface of the particles belonging to the ordered phase. Judging from the experimental results, the ordered phase was located in the vicinity of the GV membrane. Hence the depletion area due to the particles in ordered phase is overlapped with that due to the GV membrane. As a result, the particles in disordered phase freely move in the area the volume of which is larger than that of the area where the particles in ordered phase are not adjacent to GV membrane [16]. This was rationally deduced from the depletion volumes mentioned above, calculated for both origins. As illustrated in Fig 5B, $V_{dis}^{f}$ is composed of restricted regions subtracted from the areas given by the black horizontal and green vertical bars. Since the volume of the ordered phase is given by the triangular surface area of GV, $V_{dis}^{f}/V_{ord}^{f}\left(=\theta^{\prime }\;\right)$was determined by *R*s. This is in agreement with our observation that the regular arrangement of particles is located in the inner part of the GV membrane.

**Fig 6:**
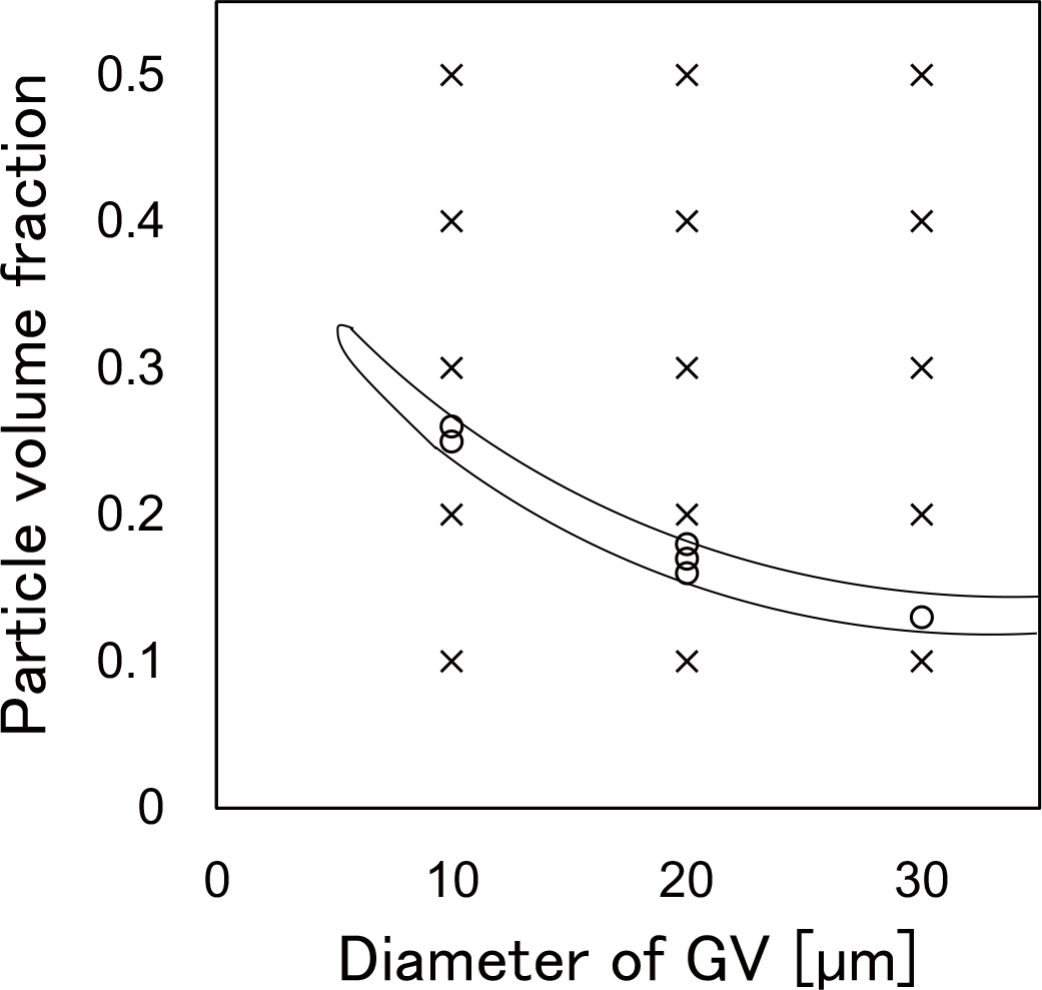
Phase diagram for emergence of the asymmetrical polyhedral GV. Phase diagram for emergence of the asymmetrical polyhedral GV in the radius of the initial spherical GV and particle volume fractions. The circles correspond to the condition requisite for the emergence of the asymmetrical polyhedral GVs where crosses were not.

When θ (affording the coexistence of the ordered and disordered phases) and *θ*′ (defined by the geometric conditions) reach the same value, *R*_*s*_ and *Φ* (for which the asymmetrical polyhedral GV is produced) are determined. Fig 6 shows the domain of *R*_*s*_ (note that the horizontal axis is 2*R*_*s*_ as diameter of GV) and *Φ*, affording *θ* / *θ*′ settled in 1.0 ± 0.1. This 10% error approximately corresponds to the experimental random error. We clearly had a solution with *R*_*s*_ = 10 μm, *Φ* = 0.16. This agrees with the experimental result demonstrating emergence of the asymmetrical polyhedral GV with *R*_*s*_ ∼ 10 μm and *Φ* ∼ 0.13.

The abovementioned argument for the model of the asymmetrical polyhedral GV indicates that the transitional characteristic formation of GVs is induced by particles exhibiting coexistence in both the disordered and ordered phases. As far as we know, the direct relationship between the coexistence of the two states of single-type particle and GV deformation had not been reported. Our experimental setup using GVs with various volume fractions of particles provides a platform for lucid detection of the crowding effect in a model cell system.

## Conclusions

In this study, we demonstrated remarkable deformation of GV containing particles with a volume fraction of ca 13 vol%. In the transformation process from the initial GVs to the stable state of GV with tubular structure, the intermediate state of asymmetrical polyhedral GV was observed reproducibly. Furthermore, characteristic coexistence of ordered and disordered phases was also observed in this GV. Consequently, we proposed a model based on the equivalence of osmotic pressures of particles in both phases and determined the free volume produced inside the GV. Considering the free volume under geometrical conditions, we obtained the conditions of GVs exhibiting the asymmetrical polyhedral configuration associated with the increase of free volume for particles. We suggest that this crowding effect involves not only volume fraction of the particles, but also the ratio of size of the particle to that of the GV. Much attention has been paid to crowding effect in both the cytosol and interior of cell organelles [17]. The current study contributes to such biological dynamics by proposing an experimental model for such crowding effects.

### Supporting Information

This material is available free of charge via the Internet at http://pubs.acs.org.

Shape changes of GV containing particles in volume fractions of 0 to several vol%. Velocity of position change for particles dispersed in spherical GV, and in orderly array in polyhedral GV. Modeling the shape of the asymmetrical polyhedral GV. Osmotic pressure of particles between the ordered and disordered phases in the equilibrium state inside the asymmetrical polyhedral GV. Geometrical derivation on free volume for particles in the ordered and disordered phases. Estimation of increase in osmotic pressure of GV.

## Methods

### Materials

1,2-Dioleoyl-sn-glycero-3-phosphocholine (DOPC) and TexasRed® tagged 1,2-dihexadecanoyl-*sn*-glycero-3-phosphoethanolamine, triethylammonium salt (TexasRed® DHPE), were purchased from Avanti Polar Lipids (AL, USA) and Invitrogen (CA, USA), respectively. The GV membrane composition was DOPC: TexasRed® DHPE = 99.7:0.3 (mol%). Polybead® carboxylate microspheres (R = 1.00 μm; 2.5 vol%; 1.05 g/cm^3^) and Fluoresbrite® yellow green carboxylate microspheres (R = 1.00 μm; 2.5 vol%; 1.05 g/cm^3^; λex, 441 nm; λem, 486 nm) were purchased from Polysciences, Inc. (PA, USA). _D_-Glucose, sucrose, and liquid paraffin were purchased from Wako Pure Chemical Industries (Osaka, Japan). The Tris-HCl buffer stock solution (100 mM, pH 7.5, 1.34 g/cm^3^) was prepared by adjusting pH of Tris (hydroxymethyl)-aminomethane (6.057 g, 50 mmol) aqueous solution to 7.5 with 1 M HCl.

### Preparation of giant vesicles (GVs)

A dispersion of microspheres (2.5 vol%) was concentrated to 10 vol% by centrifugation at 7000 × g for 5 mins (Himac CT15RE; Hitachi Koki Co., Ltd., Ibaraki, Japan). The medium for microsphere dispersion was then replaced with 100 mM Tris-HCl buffer containing _D_-glucose (0.1 M) and sucrose (0.9 M). In the present system, the polystyrene beads had a surface zeta potential of – 62 mV (Zeecom ZC-3000; Microtech Corp. Chiba, Japan). The 10 vol% microsphere dispersion (0.3 mL), liquid paraffin solution (1 mL) of DOPC (1.3 mM), and TexasRed® DHPE (3.9 × 10^−3^ mM) were homogenized using a Physcotron homogenizer (NS-310E II; Microtech Co. Ltd., Chiba, Japan) with a 4.5 μm shaft for 2 mins at 3500 rpm. The water-in-oil (w/o) emulsion dispersion (0.3 mL) was multilayered gently on the Tris-HCl buffer (100 mM, 1 mL) containing _D_-glucose (1.0 M) in a microcentrifuge tube. In order to form a lipid monolayer on the interface between oil and water, the solution was incubated for 10 mins at 4°C. After incubation, the tube was centrifuged at 18,800 × g for 30 mins at 20°C. The prepared GVs (the diameters of which were 5–25 μm) were all spherical in water and contained polystyrene beads in volume fractions of 0 vol% to 28 vol%.

### Observation of giant vesicles

As the GV precipitated due to its specific gravity, the vesicle rupture method [18] was used to prevent GV bursting. Small vesicles were obtained by sonication (for 30 mins) and filtering (100 nm) of the GV dispersion of DOPC lipids (50 mM), prepared using the film-swelling method. After the DOPC small vesicles were precipitated and ruptured on a glass plate, a thin lipid film was formed on the glass. The GV dispersion containing microspheres was then dropped on the lipid film, and specimens for microscopic observation were prepared by addition of hypertonic solution (1.1 M _D_-glucose aq.) to the GV dispersion. We used a phase contrast and fluorescence microscope (IX71, Olympus, Tokyo, Japan) and two types of confocal laser scanning microscopes: (1) CSU22 (Yokogawa Electric Corp., Tokyo, Japan) with 100× objective lens; wave length of laser, 488 nm; ca. 15.6 fps, and (2) FV1200 (Olympus, Tokyo, Japan) with 100× objective lens; wave length of laser, 559 nm; ca. 1.81 fps.

### Estimation of volume fraction

In order to simplify counting of the encapsulated microspheres, GVs containing a dispersion of microspheres (fluorescent microspheres: non-fluorescent microspheres = 1: 9, v/v) was prepared. The number *n*_*flo*_ of fluorescent microspheres in the GV was counted by florescence contrast microscopy (IX71, Olympus, Tokyo, Japan) ^10^. The volume fraction *ϕ* of inner particles estimated $\phi =\frac{n_{flo}\times v}{V_{GV}}$ where *v* and *V*_*GV*_ were the volume of microsphere and GV respectively.

### Modeling the shape of the asymmetrical polyhedral GV

To model the shape transformation of GVs, we assumed the geometrical properties, volumes, and surface areas of the initial spherical GVs (S3A Fig), asymmetrical polyhedral GVs (S3B Fig), and the GVs with tubular structures (S3C Fig) as outlined below. The initial spherical GV with radius *R*_*s*_ transformed into GV (radius; *R*_*SP*_) with a tubular structure (length, l; radius, *R*_*P*_) by osmotic stimulus of the hypertonic sugar solution. The surface area of the GV remained constant. On the other hand, the volume of the GV decreased owing to water out-flow. The volume of asymmetrical polyhedral GV, *V*_*POL*_, is same as that of the GV with tubular structure, *V*_SP_, which is smaller than the volume of initial GV, *V*_S_. Upon analyzing the microscopy images (S3 Fig), we set *R*_*S*_ = 10 μm, *R*_*SP*_ = 9.7 μm, and *R*_*P*_ = 0.5 μm (the radius of the internal colloidal particle *r*_C_) and deduced that the length of the tubular structure, l, and the volume of the GV with tubular structure (same as that of the asymmetrical polyhedral GV), *V*_*sp*_ are 23 μm and 3841 μm^3^ respectively. Since the volume of the initial GV (*V*_*s*_) was 4π*R*_*S*_^3^/3, the ratio of the volume change (*ΔV* = *V*_*S*_*−V*_*SP*_) to the initial volume of GV *ΔV/V*_*S*_ = 0.08.

The asymmetrical polyhedral GV is assumed to be a combined shape of part of an icosahedron and part of its circumscribed sphere. To obtain the number of triangular surfaces, *n*, of the icosahedron, we solved an equation $\frac{V_{S}-V_{POL}}{V_{S}}=\frac{\frac{n}{20}\left(V_{s}-V_{20}\right)}{V_{s}}=0.08\left(=\frac{\Delta V}{V_{S}}\right)$, where *V*_*20*_ is the volume of icosahedron, $V_{20}=\frac{2\sqrt{10+2\surd 5}}{3}R_{s}{}^{3}$ where *Rs* is the radius of the circumscribed sphere (corresponding to the initial sphere), and *V*_*POL*_ is the volume of the asymmetrical polyhedral GV. Since we obtained *n* ~ 4 from the equation, we modeled the asymmetrical polyhedral GV as a combined shape of four triangular surfaces of an icosahedron and a part of the circumscribed sphere (Fig 5A). This model agrees with the observed images of the GV by confocal laser scanning fluorescence microscopy (Fig 3A). When we set *R*_*S*_ *=* 5.0 μm and *R*_*SP*_ *=* 4.8 μm, we obtained *n* ∼ 5.

### Osmotic balance in the asymmetrical polyhedral GV

In general, osmotic pressure *π* is expressed by using compressibility *ζ* as follows:

$$\pi =\frac{N}{V^{f}}k_{B}T\zeta$$

where *N*, *k*_B_, and *V*^*f*^ are the number of particles, the Boltzmann constant, and the free volume for particle, respectively. The principal condition for equilibration of the ordered and disordered phases is *π*_*dis*_ = *π*_*ord*_, namely,

$$\frac{N_{dis}}{V_{dis}^{f}}\zeta_{dis}=\frac{N_{ord}}{V_{ord}^{f}}\zeta_{ord}\;\text{and}\;\frac{V_{dis}^{f}}{V_{ord}^{f}}=\frac{N_{dis}}{N_{ord}}\times \frac{\zeta_{dis}}{\zeta_{ord}}\;\text{and}\;\frac{V_{dis}^{f}}{V_{ord}^{f}}=\frac{N_{dis}}{N_{ord}}\times \frac{\zeta_{dis}}{\zeta_{ord}}$$

The subscripts, *dis* and *ord*, attached to each parameter indicate the disordered and disordered phases respectively. According to previous reports^14,15^, the values of compressibility, *ζ*_*dis*_ and *ζ*_*ord*_, are provided by the following equations using the volume fractions of particles in disordered and ordered phases, (*Φ*_*dis*_ and *Φ*_*ord*_, respectively), and a constant *ξ*:

$$\begin{array}{c} \zeta_{dis}=\frac{1+\Phi_{dis}+\Phi_{dis}^{2}-\Phi_{dis}^{3}}{{\left(1-\Phi_{dis}\right)}^{3}}\\ \zeta_{ord}=\frac{1+\Phi_{ord}+\Phi_{ord}^{2}-0.67825\Phi_{ord}^{3}-\Phi_{ord}^{4}-0.5\Phi_{ord}^{5}-6.028e^{\xi \left(7.9-3.9\xi \right)}\Phi_{ord}^{6}}{1-3\Phi_{ord}+3\Phi_{ord}^{2}-1.04305\Phi_{ord}^{3}} \end{array}$$

In this equation, ξ is the difference between the filling ratio and *Φ*_*ord*_ such that

$$\xi =\frac{\pi \sqrt{2}}{6}-\Phi_{ord}$$

Here, the value of the first term is the filling ratio of the closed packed spherical particles as follows: $\frac{4\times \frac{4}{3}\pi a^{3}}{16\sqrt{2}a^{3}}=\frac{\pi \sqrt{2}}{6}$.

First, we calculated *ζ*_*ord*_ and *N*_*ord*_. The volume fraction in the ordered phase, *Φ*_*ord*_, is assigned to be 0.545 for an ideal simple colloidal system showing coexistence of the ordered and disordered phases [19]. Thus, the compressibility *ζ*_*ord*_ equals 11.26 since $\xi =\frac{\pi \sqrt{2}}{6}-0.545=0.195$. For evaluation of *N*_*ord*_, we used the cross-section area fraction of the particles in the ordered phase along the GV membrane as *Φ*_*ord*_ = 0.545. The particles were ordered in two-dimensional hexagonal lattice as judged by the fluorescence microscopy image in Fig 4. Geometrically, as shown in S4 Fig, we evaluated the condition of the number *N*_*ord*_ of particles which are ordered in the vicinity of the triangular surface composed of flat membrane as

$$N_{ord}≦\frac{0.739\times n\times \frac{5\sqrt{3}-\sqrt{15}}{10}\times R_{s}{}^{2}}{\pi r_{c}^{2}},$$

where *n* is number of triangular surfaces.

Next, we calculated *N*_*dis*_ and *ζ*_*dis*_. The total number *N*_*c*_ of particles is given by its radius *r*_C_, the initial radius of GV *R*_S_, and the initial volume fraction *Φ* as follows:

$$N_{c}={\left(\frac{R_{s}}{r_{c}}\right)}^{3}\times \Phi$$

Thus,

$$N_{dis}=N_{c}-N_{ord}$$

On the other hand, since the reduction ratio of the volume of the asymmetrical polyhedral GV to that of the initial volume of GV, *V*_*POL*_*/V*_*S*_ (= *V*_*SP*_*/V*_*S*_), was 0.92, the volume fraction of the disordered phase is

$$\Phi_{dis}=\frac{N_{dis}\times \frac{4}{3}\pi r_{c}^{3}}{\frac{4}{3}\pi R_{S}{}^{3}\times 0.92}=\frac{N_{dis}}{0.92}{\left(\frac{r_{c}}{R_{S}}\right)}^{3}$$

We then calculated the compressibility *ζ*_*dis*_ according to the aforementioned equation. Since we can obtain *ζ*_*ord*_, *ζ*_*dis*_, *N*_*ord*_, and *N*_*dis*_ by the given *R*_*s*_, *Φ*, and *r*_*c*_, $V_{dis}^{f}/V_{ord}^{f}$ was unequivocally determined.

### Geometrical derivation on free volume for inner particles

In order to find $V_{dis}^{f}/V_{ord}^{f}$ by geometrical consideration, we first calculated the free volume for particles in the ordered phase $V_{ord}^{f}$. The particle is so hard that its center cannot approach the GV membrane in the range of its radius. This region, called exclusion region, is subtracted from the inner volume of GV affording the free volume for particles. Thus,

$$V_{ord}^{f}=0.92\times \frac{4}{3}\pi {\left(R_{s}-r_{c}\right)}^{3}$$

Next, we calculated the free volume for particles in the disordered phase $V_{dis}^{f}$. Since the vicinity of each triangular surface of membrane is occupied by the ordered phase, the particle in the disordered phase has its exclusion region. Thus $V_{dis}^{f}<V_{ord}^{f}$ should be satisfied in this situation (S5 Fig).

The volume of the exclusion region for the disordered phase *v*^*dep*^ becomes

$$v^{dep}=4\times \frac{5\sqrt{3}-\sqrt{15}}{10}R_{s}{}^{2}\times 2r_{c}$$

because the center of particles in the disordered phase cannot approach the region of ordered phases (single layered particles: thickness = 2*r*_C_) at the four triangular membranes ($\text{area per triangle surface}=\frac{5\sqrt{3}-\sqrt{15}}{10}R_{S}{}^{2}$). Therefore,

$$V_{dis}^{f}=V_{ord}^{f}-v^{dep}$$

and

$$\frac{V_{dis}^{f}}{V_{ord}^{f}}=1-\frac{v^{dep}}{V_{ord}^{f}}=1-\frac{n\times \frac{5\sqrt{3}-\sqrt{15}}{10}R_{s}{}^{2}\times 2r_{c}}{0.92\times \frac{4}{3}\pi {\left(R_{s}-r_{c}\right)}^{3}}$$

where *n* is number of triangular surfaces. *R*_*s*_ and *r*_*c*_ determines $V_{dis}^{f}/V_{ord}^{f}$ unequivocally. It is rational that *R*_*s*_ and *r*_*c*_ determines $V_{dis}^{f}/V_{ord}^{f}$ unequivocally in the case of *R*_*s*_ ≥ 20 μm, however, we have no experimental results such as the array pattern of particles in the ordered phase, the number and length of tubular membrane structure, the ratio of volume reduction, etc. Since, in the current preparation method, the GVs confining the colloidal particles at high volume fraction have the diameter less than 20 μm, the further improvement of the preparation method is needed.

## Supporting Information

S1 Fig Shape changes of GV containing particles in volume fractions of 0 to several dozen vol%. Fluorescence microscopy images of GVs with particle volume fractions of 0 vol% (a), 3 vol% (b), 23 vol% (c) (upper panels), and phase contrast microscopy images (lower panel). The GV membrane was tagged with fluorescent red. The gathered particles visible in the phase contrast microscopy image are GV internal particles. Scale bar = 10 μm. As shown in Fig a, the empty GVs (i.e., containing a volume fraction of 0 vol%) elongated along one axis and thus transitioned to a prolate conformation 120 mins after addition of 1.1 M _D_-glucose solution. Similarly, the GVs containing a volume fraction of 3–6 vol% also transitioned to a prolate conformation. In both cases, the prolate conformation remained stable till the end of the temporal observation of 120 mins following addition of the hypertonic solution. Fig b shows the GV containing a volume fraction of 3 vol% 122 mins after addition of the hypertonic solution. Meanwhile, as shown in Fig c, the GVs with a volume fraction of 23 vol% exhibited fission.
(EPS)

S2 Fig Velocity of position change for particles dispersed in spherical GV, and in orderly array in polyhedral GV. Confocal fluorescence microscopy images of a spherical GV (a) and an asymmetrical polyhedral GV (b), and histograms of velocity distribution of the particles in each of the two GVs (c). The particle velocity distribution histogram is shown in grey and red for particles in the spherical and asymmetrical polyhedral GVs, respectively. Scale bar = 10 μm. We first determined the velocities of particles dispersed in the GV in a volume fraction of 0.6 vol%, using a Nipkow disk confocal laser scanning fluorescence microscope (CSU2), to obtain images at a rate of 15.4 images/s. S2 Fig shows a representative confocal fluorescence microscopy image (Fig a). Using Image J ver. 1.47 (NIH, USA), we prepared the ultimate eroded points (UEPs) after binarization of a series of the figures. These UEPs are the centers of segmented circles on the images and correspond in position to the centers of colloidal particles. We selected a set of particles by comparison between two sequential images and calculated the velocity of particles by estimation of the displacement of UEPs. Fig c shows the velocity distribution. The mode, median, and standard deviation was 2.8 μm/s, 3.7 μm/s, and 2.7 μm/s, respectively. We next determined the velocities of particles in the orderly array during formation of the asymmetrical polyhedral GVs using confocal fluorescence microscopy images obtained at 14.3 images/s. As shown in Fig b, the particles in the asymmetrical polyhedral GV were encapsulated more densely than those in the GV with volume fraction 0.6 vol%. In order to reduce blurring of the images, we performed deconvolution using Meta Morph® (Molecular Devices, USA), and then estimated the velocities of particles by the Image J analysis mentioned above. Figs b and c show the confocal microscopy image and velocity distribution, respectively. The mode, median, and standard deviation were 0.4 μm/s, 0.09 μm/s, and 0.6 μm/s, respectively.
(EPS)

S3 FigConfiguration parameters of the observed GV shapes.(A) The initial spherical GV, (B) the asymmetrical polyhedral GV, and (C) GV with the tubular structure in stable state. The initial spherical GV with radius *R*_*s*_ transformed into GV (radius; *R*_*SP*_) with a tubular structure (length, l; radius, *R*_*P*_). Upon analyzing the microscopy images, we obtained the diameter of the initial spherical GV (2*R*_*S*_’), length of the tubular structure $\left(l^{\prime }\right)$, and the diameter of the GV in stable state (2*R*_*SP*_’); (2*R*_*S*_’, l’, 2*R*_*SP*_’) = (17 μm, 12 μm, 17μm), (21 μm, 9 μm, 20 μm), (19 μm, 13 μm, 19 μm). By the observations, *R*_*SP*_’ was equal or less *R*_*S*_’. We need to consider that the limitation of resolution of microscope is about 0.5 μm. Thus we set *R*_*S*_ = 10 μm and *R*_*SP*_ = 9.7 μm. Upon further analyzing the microscopy images, *R*_*P*_ was almost equal to the radius of the colloidal particle *r*_C_, encapsulated in the tubular structure, namely, 0.5 μm. Therefore, we deduced the length of the tubular structure as follows: $l=\frac{2}{r_{c}}\left(R_{s}^{2}-R_{sp}^{2}-\frac{1}{2}r_{C}^{2}\right)=23$ μm, which approximately agrees the obtained value by microscopy observation. We also deduced the volume of the GV with tubular structure (same as that of the asymmetrical polyhedral GV), $V_{sp}=\frac{4}{3}\pi R_{sp}^{3}+\pi R_{p}^{2}l+\frac{1}{2}\times \frac{4}{3}\pi R_{p}^{3}=3841\;{\mu \text{m}}^{3}$, neglecting the volume of the capping edge of the tubular structure. Since the volume of the initial GV (*V*_*s*_) was 4π*R*_*S*_^3^/3 = 4189 μm^3^, the ratio of the volume change (*ΔV* = *V*_*S*_*−V*_*SP*_) to the initial volume of GV *ΔV/V*_*S*_ = 0.08. Next, we calculated the difference in volume between the asymmetrical polyhedral and the initial spherical GVs geometrically. The asymmetrical polyhedral GV is assumed to be a combined shape of part of an icosahedron and part of its circumscribed sphere. The radius of the circumscribed sphere is equal to *R*_S_. The volume of icosahedron, *V*_*20*_, is generally $V_{20}=\frac{2\sqrt{10+2\surd 5}}{3}R^{3}$ where *R* is the radius of the circumscribed sphere. If the initial spherical GV transforms to symmetrical icosahedral GV, the volume ratio of the icosahedron to the circumscribed sphere, *V*_*20*_*/V*_*S*_, is $\frac{\frac{2\sqrt{10+2\surd 5}}{3}R_{s}{}^{3}}{\frac{4}{3}\pi R_{s}{}^{3}}=\frac{\sqrt{10+2\surd 5}}{2\pi }=0.605$. Then, the ratio of the volume change (*ΔV’* = *V*_*S*_*−V*_*20*_) to the initial volume of GV *ΔV’/V*_*S*_ is 0.395, which is much larger than *ΔV/V*_*S*_ (= 0.08). We next estimated the number, *n*, of triangular surfaces of the icosahedron, a part of which is combined to the circumscribed sphere for the asymmetrical polyhedral GV. The following equation involving the ratio of the volume change of the asymmetrical polyhedral GV (*V*_POL_) to *V*_S_ was used: $\frac{V_{S}-V_{POL}}{V_{S}}=\frac{\frac{n}{20}\left(V_{s}-V_{20}\right)}{V_{s}}=0.08\left(=\frac{\Delta V}{V_{S}}\right)$. Therefore we got *n* ∼ 4. Namely, we modeled the asymmetrical polyhedral GV as a combined shape of four triangular surfaces of an icosahedron and a part of the circumscribed sphere (Fig 5A). This model agrees with the observed images of the GV by confocal laser scanning fluorescence microscopy (Fig 3A). When we set *R*_*S*_ *=* 5.0 μm and *R*_*SP*_ *=* 4.8 μm, we obtained *n* ∼ 5. Although we assumed that the surface area of the initial spherical GV, *A*_*S*_, was constant during transformation of the GV (*A*_*POL*_ and *A*_*SP*_), this volume change of the initial GV to the asymmetrical polyhedral GV might change the surface area. Since the surface area of the icosahedron, *A*_20_, is generally $2\left(5\sqrt{3}-\sqrt{15}\right)R^{2}$, the ratio of the surface area per triangular surface of the icosahedron to that of the corresponding partial surface of the circumscribed sphere was estimated to be (1/20)*A*_20_/*A*_S_, using the surface area of the circumscribed sphere *A*_S_. Thus the ratio of the surface area change of the initial spherical GV to the asymmetrical polyhedral GV is: $\frac{A_{S}-A_{POL}}{A_{S}}=\frac{\frac{4}{20}(A_{s}-A_{20})}{A_{s}}=0.0475$. This ratio of reduction might indeed be ascribed to the surface area change of GV membrane deformation along the surface of the orderly arrayed colloidal particles. It is worthwhile to estimate the stability of GV with tubular structure in the stable state with respect to the change in inner pressure. We added the hypertonic sugar solution to the spherical GV and water flowed out through the GV membrane. As a result, the volume of GV decreased and its deformation was induced with its surface area constant. Although the decrease in volume was small, the inner pressure increased by the osmotic stress. In the initial state, GV with radius *R*_S_ has its volume *V*_*s*_ where $V_{s}=\frac{4}{3}\pi R_{s}^{3}$. The volume fraction of particles *Φ*_*s*_ is defined as $\Phi_{s}=\frac{N_{c}\times \frac{4}{3}\pi r_{c}^{3}}{V_{s}}$, where *N*_C_ is the number of the encapsulated particles. The free volume $V_{s}^{f}$ is given by the following equation: $V_{s}^{f}=\frac{4}{3}\pi {\left(R_{s}-r_{c}\right)}^{3}.$ Therefore we evaluated osmotic pressure $\pi_{S}=\frac{N_{C}}{V_{S}^{f}}k_{B}T\zeta_{S}$ where $V_{s}^{f}=3591,\;\Phi_{s}=0.12$, and *ζ*_*s*_ = 1.68, with *R*_*s*_ = 10 μm. In the stable state, the shape of the GV combines the sphere and the tubular structure. Since we assumed that *R*_*p*_ = *r*_*c*_ and *A*_*s*_ = *A*_*SP*_, $4\pi R_{s}^{2}=4\pi R_{sp}^{2}+2\pi lR_{p}+\frac{4\pi R_{p}^{2}}{2}$. Therefore $l=\frac{2}{r_{c}}\left(R_{s}^{2}-R_{sp}^{2}-\frac{1}{2}r_{C}^{2}\right)=23\;\mu \text{m}$, where *R*_*s*_ = 10 μm and *R*_*sp*_ = 9.7 μm. Hence the volume of the GV, *V*_*SP*_, is $V_{sp}=\frac{4}{3}\pi R_{sp}^{3}+\pi R_{p}^{2}l+\frac{\frac{4}{3}\pi R_{p}^{3}}{2}$ and the volume fraction of the particles inside the GV, *Φ*_*sp*_, becomes $\Phi_{sp}=\frac{N_{c}\times \frac{4}{3}\pi r_{c}^{3}}{V_{sp}}.$ As a result, we obtained the free volume $V_{sp}^{f}$ as follows: $V_{sp}^{f}=\frac{4}{3}\pi {\left(R_{sp}-r_{c}\right)}^{3}+\pi {\left(R_{p}-r_{c}\right)}^{2}l+\frac{\frac{4}{3}\pi {\left(R_{p}-r_{c}\right)}^{3}}{2}=\frac{4}{3}\pi {\left(R_{sp}-r_{c}\right)}^{3}$ because *R*_*p*_ = *r*_*c*_. Hence, we calculated the osmotic pressure $\pi_{sp}=\frac{N_{C}}{V_{sp}^{f}}k_{B}T\zeta_{SP}$ where $V_{sp}^{f}=3262,\;\Phi_{sp}=0.13$, and *ζ*_*sp*_ = 1.77, with *R*_*s*_ = 9.7 μm and *N*_*c*_ = 980. Finally, we obtained *π*_*sp*_ / *π*_*s*_ = 1.16. This slight increase of the osmotic pressure is admissible in terms of induction of the tubular structure formation of the GV driven by addition of the hypertonic sugar solution.(EPS)

S4 FigSchematic illustration of particles (radius, *r*_C_ = *a* –Δ*a*) in the ordered phase.Generally, the area fraction of the closed packed spherical particles (the radius of which is *a*) is the ratio of the area of summed partial circles of the particle to that of the triangular region composed of the particle centers: $\frac{\frac{\pi a^{2}}{2}}{\sqrt{3}a^{2}}.$ This is the filling ratio of the closed packed spherical particles. The particles with *Φ*_*ord*_ = 0.545 tend to be aligned with a gap, *Δa* (= *a*–*r*_C_). Then the area fraction was: $\frac{\frac{\pi }{2}{\left(a-\Delta a\right)}^{2}}{\sqrt{3}a^{2}}=\frac{\pi }{2\sqrt{3}}{\left(1-\frac{\Delta a}{a}\right)}^{2}=\frac{\pi }{2\sqrt{3}}\eta^{2}$, where *η* = 1 – Δ*a*/*a*. We supposed that the ordered phase consists of face-centered cubic with the filling ratio as mentioned before. Therefore, the particle with radius *r*_c_ (= *a* – Δ*a*) affords its volume fraction as $\frac{\pi \sqrt{2}}{6}{\left(1-\frac{\Delta a}{a}\right)}^{3}=\frac{\pi \sqrt{2}}{6}\eta^{3}$. Thus we estimated the reduction factor *η*^3^ for the colloidal packing as follows: Here the volume fraction of particles in closed packed situation was $\frac{4\times \frac{4}{3}\pi a^{3}}{16\sqrt{2}a^{3}}=\frac{\pi \sqrt{2}}{6}=0.740$. Therefore, we deduced *η* with *Φ*_*ord*_ = 0.545 as $\eta^{3}=\frac{0.545}{0.740}=0.736$. The one-dimensional reduction factor reflecting the apparent colloidal ordering was $\eta =\sqrt[{3}]{0.736}=0.903.$ This estimation enabled us to suggest that the value of *η* is the ratio between twice the radius *r*_C_ and distance for center of mass for colloids. In fact, this value agrees well with our experimental results demonstrating 1.1 μm for *r*_C_ = 0.5 μm. Accordingly, we proceeded to the estimation of the area fraction: $\frac{\pi }{2\sqrt{3}}\eta^{2}=0.739.$ Consequently, we evaluated the condition of the number *N*_*ord*_ of particles which are ordered in the vicinity of four triangular surface composed of flat membrane ($\text{area per triangular surface}\;=\;\frac{5\sqrt{3}-\sqrt{15}}{10}R_{S}{}^{2}$). The expression of inequality for the integer value is $N_{ord}≦\frac{0.739\times n\times \frac{5\sqrt{3}-\sqrt{15}}{10}\times R_{s}{}^{2}}{\pi r_{c}^{2}},$ where *n* is number of triangular surfaces.(EPS)

S5 Fig Schematic illustration of exclusion regions for particles in the ordered and disordered phases. The particle is so hard that its center cannot approach the GV membrane in the range of its radius. In addition The center of particles in the disordered phase cannot approach the region of ordered phases (single layered particles: thickness = 2*r*_C_).
(EPS)
